# Apolipoprotein A-I infiltration in rheumatoid arthritis synovial tissue: a control mechanism of cytokine production?

**DOI:** 10.1186/ar1443

**Published:** 2004-10-06

**Authors:** Barry Bresnihan, Martina Gogarty, Oliver FitzGerald, Jean-Michel Dayer, Danielle Burger

**Affiliations:** 1 Department of Rheumatology, St Vincents University Hospital, Dublin, Ireland; 2 Service of Immunology and Allergy, Faculty of Medicine, Geneva, Switzerland

**Keywords:** apolipoprotein A-I, cytokines, inflammation, rheumatoid arthritis, synovium

## Abstract

The production of tumor necrosis factor α (TNF-α) and interleukin-1β (IL-1β) by monocytes is strongly induced by direct contact with stimulated T lymphocytes, and this mechanism may be critical in the pathogenesis of rheumatoid arthritis (RA). Apolipoprotein A-I (apoA-I) blocks contact-mediated activation of monocytes, causing inhibition of TNF-α and IL-1β production. This study examined the hypothesis that apoA-I may have a regulatory role at sites of macrophage activation by T lymphocytes in inflamed RA synovial tissue. Synovial tissue samples were obtained after arthroscopy from patients with early untreated RA or treated RA and from normal subjects. As determined by immunohistochemistry, apoA-I was consistently present in inflamed synovial tissue that contained infiltrating T cells and macrophages, but it was absent from noninflamed tissue samples obtained from treated patients and from normal subjects. ApoA-I staining was abundant in the perivascular areas and extended in a halo-like pattern to the surrounding cellular infiltrate. C-reactive protein and serum amyloid A were not detected in the same perivascular areas of inflamed tissues. The abundant presence of apoA-I in the perivascular cellular infiltrates of inflamed RA synovial tissue extends the observations *in vitro *that showed that apoA-I can modify contact-mediated macrophage production of TNF-α and IL-1β. ApoA-I was not present in synovium from patients in apparent remission, suggesting that it has a specific role during phases of disease activity. These findings support the suggestion that the biologic properties of apoA-I, about which knowledge is newly emerging, include anti-inflammatory activities and therefore have important implications for the treatment of chronic inflammatory diseases.

## Introduction

Inflammation is a critical host-defense mechanism. One of its functions is to direct plasma factors and immunoinflammatory cells to lesions in order to eradicate infection and facilitate tissue repair. In many chronic inflammatory diseases, infiltration of the target tissue by blood-derived cells precedes tissue damage. For example, it is believed that in rheumatoid arthritis (RA), the initial cellular event in synovial tissue is proliferation of fibroblast-like synoviocytes, which release chemokines that contribute to the recruitment of inflammatory cells, including monocytes and lymphocytes [1]. It has been proposed that the first cells to infiltrate synovial tissue are T lymphocytes, suggesting that they have an important role in pathogenesis. We previously showed that stimulated T cells induced pathological effects through direct cellular contact with monocyte–macrophages, causing the abundant production of interleukin-1β (IL-1β) and tumor necrosis factor α (TNF-α). This observation has been confirmed by others (for review see [2]). The unregulated production of IL-1β and TNF-α in RA has been recognized for several years, and their role in the pathophysiology has been confirmed by the demonstration that targeted blockade improves patients' clinical status [3,4].

We therefore postulate that contact-mediated cytokine production is highly relevant to the pathogenesis and the maintenance of chronic inflammation in diseases such as RA. Regulating a potent mechanism that induces both IL-1β and TNF-α may be important in maintaining a low level of monocyte activation within the bloodstream. We recently identified apolipoprotein A-I (apoA-I) as a specific inhibitor of contact-mediated activation of monocytes [5]. ApoA-I is a 'negative acute-phase protein' and the principal protein of high-density lipoproteins (HDLs). Variations of apoA-I concentration have been observed in several inflammatory diseases. In RA, the levels of circulating apoA-I and HDL cholesterol in untreated patients are lower than in normal controls [6-8]. In contrast, apoA-I levels were increased in the synovial fluid of patients with RA [9], although these were still only one-tenth those in plasma. The elevation of apoA-I levels in the synovial fluid of untreated patients with RA was accompanied by increased cholesterol levels, suggesting infiltration of HDL particles in the inflamed joint. In this study, we examined synovial tissue from patients with active RA in order to determine if apoA-I infiltration had occurred at sites of contact between T lymphocytes and macrophages.

## Materials and methods

### Synovial tissue samples

Synovial biopsies were obtained from the knee joints after arthroscopy in patients diagnosed with RA, who had all given their informed consent. Normal synovium was obtained from a patient without arthritis who was having a leg amputated. Arthroscopy and biopsy were performed under local anesthesia using a 2.7-mm Storz arthroscope and a 1.5-mm grasping forceps. The sampled tissue was immediately embedded in Tissue-Tek^® ^OCT compound (Sakura, Zoeterwoude, the Netherlands) and snap frozen in liquid nitrogen.

### Monoclonal antibodies

All antibodies used were murine antihuman monoclonal antibodies (antibodies were diluted in PBS; anti-apoA-I contained 0.6 M sodium chloride); anti-apoA-I, type 2 (Calbiochem-Novabiochem Corporation, Darmstadt, Germany), was used at 1/3000 dilution; anti-C-reactive protein (CRP), clone CRP-8 (Sigma Chemicals, St Louis, MO, USA), at 1/200 dilution; anti-Von Willebrand factor/factor VIII-related antigen (FVIII), clone F8/86 (DAKO, Glostrup, Denmark), at 1/50 dilution; and anti-acute-phase serum amyloid A (A-SAA) (gift from Dr AS Whitehead, Philadelphia, PA, USA), at 1/1200. Isotype-matched murine IgG1 (DAKO) was used at the same concentration as each of the primary antibodies.

### Immunohistochemistry

Synovial tissue sections were cut at 7 μm and mounted on slides coated with 3-aminopropyltriethoxy-silane (Sigma). Slides were air-dried overnight, wrapped in foil, and stored at -80°C. A standard three-stage immunoperoxidase technique was used, with a Peroxidase VECTASTAIN^® ^Elite ABC kit (Vector Laboratories, Burlingame, CA, USA). Slides were removed from the -80°C freezer and allowed to thaw at room temperature for 20 minutes. Sections were fixed in acetone for 10 minutes and with normal horse serum (VECTASTAIN^® ^Elite ABC kit) for 15 minutes. The relevant primary antibody was added to sections for 1 hour at room temperature. Sections were washed and incubated with PBS for 5 minutes. Anti-mouse IgG secondary antibody (VECTASTAIN^® ^Elite ABC kit) was added for 30 minutes and the ABC solution (VECTASTAIN^® ^Elite ABC kit) was added to sections for 30 minutes. Sections were treated with 3% hydrogen peroxide for 7 minutes, washed in distilled water for 1 minute, and incubated in PBS for 5 minutes, followed by the addition of 3,3'-diaminobenzidine (Sigma) for 12 minutes. The chromogenic reaction was stopped by immersion in water. Sections were counterstained in Mayer's hemalum, dehydrated in alcohol, cleared in xylene, and mounted in DPX (BDH, Poole, UK).

## Results

The demographic and clinical details of the patients studied are outlined in Table 1. Synovial tissue samples from eight patients with active RA were selected. The mean duration of disease was 19 (range 1–48) months, he mean swollen joint count was 20 (range 10–36), and the mean CRP level was 12.3 (range <3 to 22)mg/L. Six patients were receiving nonsteroidal anti-inflammatory drugs at the time of synovial biopsy. Two were receiving a disease-modifying anti-rheumatic drug, methotrexate, 15 mg/week in both cases. Two were receiving prednisolone, 10 mg/day. None had received an intra-articular corticosteroid injection to the biopsied knee joint. Synovial tissue was also obtained from two patients with quiescent RA (no swollen joints, CRP <3 mg/L) and from one patient who was unaffected by arthritis. Both patients with quiescent RA were receiving methotrexate, 7.5 mg/week.

**Table 1:** Demographic and clinical details of patients with active rheumatoid arthritis

Total no. of patients	8
Mean duration of disease (range)	19 (1–48) months
Mean no. of swollen joints (range)	20 (10–36)
Mean C-reactive protein (range)	12.3 (0–22)mg/dL
No. of patients receiving:	
NSAIDs	6
DMARDs (MTX 15 mg/wk)	2
Prednisolone	2

DMARD, disease-modifying antirheumatic drug; MTX, methotrexate; NSAID, nonsteroidal anti-inflammatory drug.

**Figure 1:**
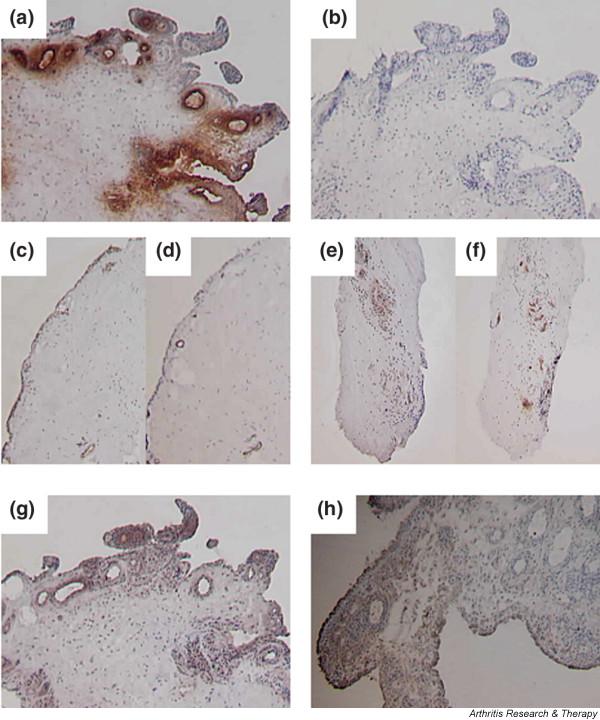
Apolipoprotein A-I (apoA-I) is localized in the perivascular region of the inflamed synovium. **(a) **Active rheumatoid arthritis (RA) synovium stained with anti-apoA-I; **(b) **active RA synovium stained with isotype-matched negative control; **(c) **normal synovium stained with anti-apoA-I; **(d) **normal synovium stained with anti-factor VIII; **(e) **remission RA synovium stained with anti-apoA-I; **(f) **remission RA synovium stained with anti-factor VIII; **(g) **active RA synovium stained with anti-C-reactive protein; **(h) **active RA synovium stained with antibody against serum amyloid A.

All synovial tissue sections from the eight patients with active RA showed prominent blood vessels and perivascular cellular infiltration. Specific apoA-I staining was present in all samples. The immunohistologic appearances were consistent, and included prominent endothelial apoA-I staining of most blood vessels (Fig. 1a). The vessels were surrounded by a confined area of intense staining that was consistent with extravasation of apoA-I within the perivascular cell infiltrate. No staining was observed in the negative control tissue sections (Fig. 1b). In tissue samples obtained from patients with RA that were in apparent remission, only faint vascular and perivascular apoA-I staining was present (Fig. 1e), even though the sections contained blood vessels that were easily identified (Fig. 1f). As expected, the cellular infiltrate in these sections was less intense. There was no perivascular apoA-I staining in the synovial tissue sample obtained from the knee joint unaffected by arthritis (Fig. 1c). Contrary to the abundant presence of perivascular apoA-I staining in tissue sections obtained from patients with active RA, there was no evidence of perivascular CRP or A-SAA. Tissue samples from three patients were studied for the presence of perivascular CRP. The serum CRP levels were elevated in all three at the time of biopsy (11–20 mg/L). Faint CRP staining of endothelial cells was observed (Fig. 1g). Tissue samples from five patients were studied for the presence of perivascular A-SAA. As expected, A-SAA staining was demonstrated in lining layer cells but not within the perivascular infiltrate (Fig. 1h).

## Discussion

The most salient observation from this study was apoA-I infiltration in inflamed synovial tissue and its retention in perivascular regions, where T lymphocytes and macrophages accumulate. The localization of positive acute-phase proteins, such as CRP and A-SAA, was different from that of apoA-I: only faint staining, limited to vascular endothelium, was observed for CRP, and A-SAA was observed in lining layer cells, which are a known source of local synthesis [10].

We have previously shown apoA-I to inhibit the production of both IL-1β and TNF-α in monocytes activated by direct contact with stimulated T cells. This mechanism may have a role in regulating monocyte activation in the bloodstream [5]. This study demonstrated that apoA-I infiltrated perivascular regions of the synovium where A-SAA, which can dissociate apoA-I from HDLs [11], and CRP were absent. The perivascular localization of apoA-I suggests that it could have an inhibitory role in zones where T lymphocytes are in close contact with monocyte–macrophages, with a tendency to form 'lymphoid microstructures' [12]. The absence of A-SAA suggests that it is unlikely to restrict the inhibitory activity of apoA-I in the contact-mediated induction of IL-1β and TNF-α production in tissue [13]. To overcome apoA-I inhibition, A-SAA would be expected to localize in the same area. Since apoA-I is virtually absent from the synovial tissue of patients with inactive RA (Fig. 1c), its presence in actively inflamed tissue suggests that its infiltration during a flare-up may represent a physiologic mechanism that inhibits proinflammatory cytokine production and limits disease recurrence. The transient infiltration of apoA-I may also explain why RA, like many other chronic inflammatory diseases, characteristically presents as a relapsing–remitting disease in many patients. During phases of RA associated with joint damage, the inhibitory effects of apoA-I on the destructive mechanisms may not be sufficiently potent.

In RA, variations of apoA-I concentrations were observed in plasma, where it was decreased, and in synovial fluid, where it was increased [6-9]. The elevation of apoA-I levels in synovial fluid of RA patients correlated with a rise in cholesterol, suggesting infiltration of HDL particles into the inflamed joint. Similarly, active juvenile RA was associated with reduced HDL blood levels and a significant decrease in apoA-I concentration in plasma [14]. These studies suggest that variations of apoA-I levels may inversely correlate with disease activity. The observation that apoA-I can infiltrate and be retained at the inflammatory site suggests that apoA-I may inhibit the local triggering of IL-1β and TNF-α release by monocyte–macrophages that are in direct contact with stimulated T cells in these areas [15].

## Conclusion

In conclusion, the localization of apoA-I in inflamed synovium suggests that it can locally inhibit the production of proinflammatory cytokines by monocyte–macrophages upon direct contact with stimulated T cells. Thus, it is possible that after immune cell infiltration, formation of lymphoid-like microstructures, and the proliferation of blood vessels that resemble high-endothelial venules, inhibitory plasma components may infiltrate the developing inflammatory lesion. ApoA-I that binds surface factors on stimulated T cells is retained in the perivascular regions, where it may limit contact-mediated cytokine induction in monocyte–macrophages [5] and inhibit critical pathways associated with disease exacerbation. The alterations in apoA-I infiltration may also explain fluctuations of disease activity. The finding that apoA-I can infiltrate inflamed tissue, together with its newly emerging anti-inflammatory properties, may have important implications for treatment in chronic inflammatory diseases.

## Competing interests

The authors declare that they have no competing interests.

## Author contributions

BB and OF cared for the patients included in this study and supervised arthroscopy and biopsy procedures.

MG carried out the histochemical study.

BB, JMD, and DB conceived of the study and participated in its design and coordination.

All authors read and approved the final manuscript.

## Abbreviations

apo A-I = apolipoprotein A-I; A-SAA = acute-phase serum amyloid A; CRP = C-reactive protein; HDL = high-density lipoprotein; IL-1β = interleukin-1β; PBS = phosphate-buffered saline; RA = rheumatoid arthritis; TNF-α = tumor necrosis factor α.
